# Laboratory antimicrobial resistance surveillance: extend spectrum beta lactamase (ESBL) producing *E. coli*

**DOI:** 10.1186/1753-6561-5-S6-P135

**Published:** 2011-06-29

**Authors:** S Mobasherizadeh, S Jalalpoor, A Ebneshahidi, L Ghaedi

**Affiliations:** 1 Isfahan University of Medical Sciences, Isfahan, Iran, Islamic Republic of; 2 2. Islamic Azad University Shahreza Branch, Isfahan, Iran, Islamic Republic of; 3 Sadi Hospital, Isfahan, Iran, Islamic Republic of

## Introduction / objectives

This study aims to investigate the prevalence of ESBL producing *E.coli* in urinary tract nosocomial and community acquired infections of Isfahan selected hospitals.

## Methods

The present study was performed at four tertiary care hospitals in Isfahan, Iran. During a 14 month period (Jun, 7^th^,2008 to July,6^th^ ,2010).690 of E.coli isolated from urinary tract infection were studied. 425 of E.coli isolated from community acquired and 265 isolated from nosocomial urinary tract infection were evaluated. Standard microbiological methods were performed(according to CLSI 2006). In order to validate the extended-spectrum beta-lactamases (ESBLs) producing strains were used by disk diffusion method. The collected data was analyzed thorough whonet 5.6 software.

## Results

The prevalence of ESBLs producing E.coli isolated from community acquired urinary tract infection came out to be 17% and 58% for urinary tract nosocomial infection respectively(P<0.001).The antibiotic resistance rates of isolated in nosocomial and community UTIs were 94.9% and 84.4% to ampicillin.(P<0.01),59.4% and 19.7% to ceftazidime, 64.2% and 19.8% to cefotaxime, 62.5% and 12.2% to ceftizoxime 60% and 18.6% to gentamicin.(P<0.001), 17% and 8.2% to amikacin , and 40.8% to Nalidixic acid, 23.1% and 10.2% to nitrofurantoin,47.5% and 31.4% to ciprofloxacin and 84.4% and 60.1% to trimethoprim/sulfamethoxazole (p<0.005).

## Conclusion

Establish systems for monitoring antimicrobial resistance in hospitals and the community and link these findings to resistance and disease surveillance data is fundamental to developing treatment guidelines accurately and to assessing the effectiveness of interventions appropriately.

## Disclosure of interest

None declared.

